# The Effect of a TLR4 Agonist/Cationic Liposome Adjuvant on Varicella-Zoster Virus Glycoprotein E Vaccine Efficacy: Antigen Presentation, Uptake, and Delivery to Lymph Nodes

**DOI:** 10.3390/pharmaceutics13030390

**Published:** 2021-03-15

**Authors:** Seo Ri Wui, Ara Ko, Ji In Ryu, Eojin Sim, Soo Jeong Lim, Shin Ae Park, Kwang Sung Kim, Ha Kim, Hyewon Youn, Na Gyong Lee

**Affiliations:** 1 Department of Integrated Bioscience and Biotechnology, Sejong University, Seoul 05006, Korea; ehwls4144@naver.com (S.R.W.); jellyz47@naver.com (A.K.); bycal87@naver.com (J.I.R.); simej1003@naver.com (E.S.); sjlim@sejong.ac.kr (S.J.L.); black@eyegene.co.kr (K.S.K.); 2 R & D Center, EyeGene, Goyang 10551, Korea; gold@eyegene.co.kr; 3 Cancer Research Institute, College of Medicine, Seoul National University, Seoul 03080, Korea; kh881210@hanmail.net (H.K.); hwyoun@snu.ac.kr (H.Y.); 4 Department of Nuclear Medicine, Cancer Imaging Center, Seoul National University Hospital, Seoul 03080, Korea

**Keywords:** antigen delivery to lymph nodes, cationic liposomes, cellular antigen uptake, CIA09, TLR4 agonist de-*O*-acylated lipooligosaccharides, vaccine adjuvant, VZV gE antigen

## Abstract

Adjuvant CIA09, composed of 1,2-dioleoyl-3-trimethylammonium-propane (DOTAP)-based cationic liposomes and the toll-like receptor 4 agonist de-*O*-acylated lipooligosaccharide (dLOS), has been shown to enhance antibody and cellular immune responses to varicella-zoster virus (VZV) glycoprotein E (gE), recombinant tuberculosis vaccine antigen, and inactivated Japanese encephalitis vaccine. In this study, we investigated its modes of action using VZV gE as a model antigen. Liposomes adsorbed gE and cooperatively with dLOS promoted endocytosis-mediated cellular uptake of gE by mouse dendritic cells in vitro. CIA09 increased the stability and cellular uptake of the antigen at the muscle site of injection, and induced immune cell recruitment and cytokine and chemokine production, which led to efficient antigen delivery to draining lymph nodes. Mouse bone marrow-derived dendritic cells, pulsed with CIA09-adjuvanted gE, efficiently presented gE to antigen-specific T cells, inducing Th1-type biased immunity, as shown by high IFN-γ production. The data indicate that liposomes and dLOS cooperate in the adjuvant activity of CIA09 by promoting antigen uptake and delivery to lymph nodes as well as antigen presentation to T cells.

## 1. Introduction

Adjuvants are an important component of many vaccines, especially recombinant subunit vaccines. Vehicle type adjuvants, which act as antigen carriers, include alum, oil-in-water emulsions, and liposomes [1,2,3]. They serve as an antigen depot, increase the stability of the antigen, enhance uptake by antigen-presenting cells (APCs), and facilitate delivery to lymph nodes [2,3,4,5]. Immunomodulatory adjuvants directly activate immune cells, induce cytokines and chemokines, and recruit immune cells [2,5]. They are typically ligands for cellular pattern recognition receptors (PRRs), including toll-like receptors (TLRs), C-type lectin receptors (CLRs), nucleotide-binding oligomerization domain (NOD)-like receptors (NLRs), retinoic acid-inducible gene I (RIG-I)-like receptors (RLRs), and cytosolic dsDNA sensors (CDSs) [6,7,8,9,10,11]. These ligands mimic pathogen or damage-associated molecular patterns (PAMPs/DAMPs) to recognize PRRs and induce the rapid activation of innate immune responses, leading to adaptive immunity [6,7,8,9,10,11]. Recently designed adjuvant formulations, containing two or more adjuvants with different, cooperative modes of action, provide high adjuvant activity, optimizing the efficacy of vaccines [12,13].

Liposomes, which are biocompatible, biodegradable, non-toxic, and have long been used as drug delivery systems, also function as adjuvants for human vaccines [14,15,16,17,18]. Liposomal adjuvants serve as persistent depots of vaccine antigens at the site of injection (SOI), facilitate antigen delivery to lymph nodes, and increase antigen uptake and processing by APCs [14,15,16,17,18]. Cationic liposomes, which bind electrostatically to negatively charged protein antigens and cell surface membranes, are particularly effective adjuvants [19,20,21]. Unlike neutral or anionic liposomes, cationic liposomes directly activate immune cells, such as dendritic cells (DCs) and macrophages [22,23,24,25]. A cationic liposome adjuvant, based on 1,2-dioleoyl-3-trimethylammonium-propane (DOTAP), enhances humoral and cell-mediated immune responses to antigens produced in vitro or in vivo in mice [26,27,28]; it also induces activation of the extracellular-signal-regulated kinase (ERK) pathway and the synthesis of chemokines in mouse bone marrow-derived DCs (BMDCs) [29]. However, cationic liposomes require other immune stimulants for optimal adjuvant activity [24,30]; for example, the liposome-based adjuvant CAF01 includes the cationic lipid dimethyldioctadecylammonium (DDA) and synthetic trehalose-6,6-dibehenate (TDB) [31,32]. CAF01 induces strong humoral and cellular immune responses and is a potential adjuvant for a tuberculosis vaccine, having elicited protective immunity against a *Mycobacterium tuberculosis* challenge in mouse and human clinical trials [33,34,35].

De-*O*-acylated lipooligosaccharide (dLOS) is a TLR4 agonist derived from *Escherichia coli* lipopolysaccharide (LPS) with an additional short carbohydrate moiety [36,37]. It induces the secretion of cytokines from murine peritoneal macrophages similarly to MPL but with more potent activation of human monocytes and DCs [37]. dLOS in combination with aluminum hydroxide (designated CIA06) has adjuvant activity in several viral and bacterial vaccines [38,39,40,41,42] and performed well in a phase I clinical study of a CIA06-adjuvanted human papillomavirus (HPV) virus-like particle (VLP) vaccine (unpublished data). We developed cationic liposome-based adjuvant CIA09, composed of DOTAP-based cationic liposomes and dLOS, which enhances antibody and cell-mediated immune responses to recombinant tuberculosis antigens, inactivated Japanese encephalitis vaccine (JEV), and recombinant varicella-zoster virus (VZV) glycoprotein E (gE) antigen [42,43,44]. CIA09 is particularly effective in eliciting a Th1-type biased response, as determined by interferon-γ (IFN-γ) cytokine production, compared with CIA06 [42,43,44]. Using VZV gE as the model antigen, we investigated the mechanism of action of CIA09 and demonstrate here that liposomes and dLOS cooperatively promote (i) the immunogenicity of VZV gE antigen by increasing the antigen stability, (ii) antigen uptake at the site of injection (SOI), (iii) the recruitment of immune cells, (iv) antigen delivery to the lymph nodes, and v) antigen presentation by APCs to T cells.

## 2. Materials and Methods

### 2.1. Experimental Animals

BALB/c and C57BL/6 mice used for experiments were purchased from SLC (Hamamatsu, Japan) or Orient Bio (Orient Bio, Gyeonggi-do, Korea). Mice were housed in a temperature- and humidity-controlled chamber with a 12-h light/dark cycle and provided with free access to food and water. Mice were anesthetized with an intraperitoneal injection of a ketamine/xylazine mixture before being used for experiments or sacrificed for tissue samples.

### 2.2. Materials

The Madin-Darby canine kidney (MDCK) cell line and the J774A.1 mouse monocyte/macrophage cell line were obtained from ATCC (Manassas, VA, USA), while the DC2.4 mouse immature dendritic cell line was kindly provided by Prof. I. Rhee of Sejong University, Republic of Korea. Two phospholipids, DOTAP and 1,2-dimyristoyl-sn-glycero-3-phosphocholine (DMPC), were purchased from Avanti Polar Lipids (Alabaster, AL, USA) and/or NOF (Tokyo, Japan). NBD-labeled DOTAP was obtained from Avanti Polar Lipids, whereas 1,1′-dioctadecyl-3,3,3′,3′-tetramethylindotricarbocyanine iodide (DiR) and DAPI were from Invitrogen (Carlsbad, CA, USA) and Lonza (Basel, Switzerland), respectively. Cytochalasin D (actin polymerization inhibitor), chlorpromazine (clathrin-mediated endocytosis inhibitor), and genistein (caveolae-mediated endocytosis inhibitor) were purchased from Sigma-Aldrich (St. Louis, MO, USA). Cell culture media and antibiotics were obtained from Welgene (Gyeongsangbuk-do, Korea), whereas fetal bovine serum (FBS) was from Gibco/Invitrogen (Grand Island, NY, USA). The IFN-γ cytokine ELISA kit and the multiplex assay kit using Luminex^®^ were from R&D Systems (Minneapolis, MN, USA), while interleukin-5 (IL-5) and monocyte chemoattractant protein-1 (MCP-1) ELISA kits were from BD Bioscience (San Jose, CA, USA). Anti-mouse CD16/CD32 Ab, anti–CD11b-FITC, anti-CD11c-FITC, anti-Ly6C-PE, anti-F4/80-PE, anti-Ly6G-PE, anti-MHCII-PE, anti-CD3ɛ-PE, anti-CD11b-PE-Cy7, IC fixation buffer, and permeabilization buffer were purchased from eBioscience (San Diego, CA, USA) or BioLegend (San Diego, CA, USA).

### 2.3. Preparation of dLOS and Recombinant VZV gE Antigen

The TLR4 agonist dLOS was isolated from an *E. coli* strain that expresses LPS lacking *O*-polysaccharides and prepared as previously described [36,37], visualized on a silver-stained SDS-polyacrylamide gel, and quantified using the 2-keto-3-deoxyoctonate assay [45].

Recombinant VZV gE antigen was expressed in Chinese hamster ovary (CHO) cells and purified as previously described by Wui et al. [44]. Protein was quantified with the Bradford assay kit, while the endotoxin contents in gE preparations were determined using the Endosafe-PTS test system (Charles River Laboratories, Wilmington, MA, USA) and confirmed to be lower than 1 EU/μg protein. To prepare fluorescently labeled gE antigen, recombinant VZV gE was conjugated with fluorescent dyes using an Alexa Fluor (AF) 488 or 647 protein labeling kit (Invitrogen) or HiLyte^TM^ Fluor 647 amine (AnaSpec, Fremont, CA, USA) according to the manufacturer’s instructions.

### 2.4. Preparation and Characterization of Cationic Liposomes

#### 2.4.1. Liposome Preparation

Liposomes were prepared with cationic lipid DOTAP and neutral lipid DMPC by a dry cake method as previously described by Wui et al. [44]. Briefly, DOTAP and DMPC were each dissolved in tertiary-butyl alcohol and mixed at a molar ratio of 1:1. The lipid mixture was lyophilized overnight using a freeze-dryer (Lyoph-Pride; Ilshin BioBase, Gyeonggi-do, Korea). The dry cakes were hydrated with a 10% sucrose solution to a final concentration of 2 mg lipid/mL and homogenized using a microfluidizer (Avestin, Ottawa, ON, Canada). Liposome preparations were aliquoted in sealed glass vials, freeze-dried, and stored at −20 °C. For experiments, freeze-dried liposomes were reconstituted with deionized water to the original volume. Fluorescently labeled liposomes were made by adding 1% DiR- or NBD-labeled DMPC to the phospholipid mixtures (*w*/*w*) before lyophilization. CIA09 was prepared by combining liposomes with dLOS at various ratios.

#### 2.4.2. Measurement of Particle Size and Zeta Potential of Liposomes

The particle size and polydispersity index (PDI) of liposomes were determined by dynamic light scattering (DLS) using a Zetasizer Nano ZSP (Malvern Instruments, Worcestershire, UK). Liposome powder was rehydrated with deionized water to its original volume and further diluted tenfold with a 10% sucrose solution. Samples were analyzed at a detection angle of 173° and a recording temperature of 25 °C. The zeta potential of liposomes was measured by laser doppler microelectrophoresis on the Zetasizer Nano ZSP. Malvern Zetasizer DTS software (version 7.12) was used for data acquisition and analysis. All measurements were carried out in triplicate.

#### 2.4.3. Morphology

The morphology of liposomes was determined by cryogenic transmission electron microscopy (Cryo-TEM). Lyophilized liposome powder was rehydrated with deionized water, and 3 μL of the liposome preparation was placed on a lacey carbon film 200-mesh copper grid and vitrified semi-automatically using a Vitrobot™ (FEI, Hillsboro, OR, USA). Vitrified samples were maintained at liquid nitrogen temperatures during sample transfer and imaged with a Tecnai G2 Spirit Twin electron microscope (FEI) at 120 keV.

#### 2.4.4. Cytotoxicity Assay

The cytotoxicity of liposomes was determined by the MTT assay in MDCK and DC2.4 cells. Cells were seeded in DMEM or RPMI1640 supplemented with 10% FBS at a density of 2 × 10^5^ cells/mL in a 96-well plate and cultured overnight. Cells were treated with liposomes (25, 50, 100, or 200 μg/mL) in the presence or absence of dLOS (0.5, 1, 2, or 4 μg/mL) for 24 h. The next day, CCK-8 solution (Dojindo, Tokyo, Japan) was added to the culture, and cells were incubated for 2 h. Optical density at 450 nm was measured using a microplate reader (Infinit M200; Tecan, Mannedorf, Switzerland). All assays were performed in triplicate. Cell viability relative to that of control cells was calculated as: cell viability (%) = ((OD_450_ of test cells − OD_450_ of blank well)/(OD_450_ of media control cells − OD_450_ of blank well)) × 100.

### 2.5. Measurement of gE Antigen Adsorption on Liposomes

Recombinant VZV gE antigen (10 μg) was mixed with liposomes (200 μg) in the presence or absence of dLOS (4 μg) in a total volume of 200 μL. The mixture was ultracentrifuged at 100,000× *g* at 4 °C for 1 h. The supernatant was removed, and the pellet was dissolved in the original volume of phosphate-buffered saline (PBS, pH 7.2). gE protein in the supernatant and pellet was resolved on SDS-PAGE gels and silver-stained. The intensity of the protein bands on gels was determined by image processing of the bands using Image J program (NIH, Bethesda, MD, USA).

### 2.6. Determination of Cellular Uptake of gE Antigen

#### 2.6.1. Imaging of Cellular Antigen-Uptake by Confocal Microscopy

DC2.4 cells were seeded at a density of 2 × 10^5^ cells/mL in 4-well cell culture slides and cultured overnight. Cells were incubated with fluorescently labeled gE (5 µg/mL), alone or in combination with dLOS (3 µg/mL), NBD-labeled liposomes (100 µg/mL), or both (CIA09) for 4 h. Cells were washed, fixed, and stained with DAPI. The slides were washed, mounted with antifade mounting medium (Invitrogen), and observed under a confocal microscope (Carl Zeiss, Oberkochen, Germany).

For live-cell image analysis, DC2.4 cells were seeded at a density of 1 × 10^4^ cells/well in a Scar™ Block confocal dish (SPL, Gyeonggi-do, Korea) and cultured overnight. Cells were treated with fluorescently labeled gE (5 µg/mL), alone or in combination with dLOS (2 µg/mL), DiR-labeled liposomes (100 µg/mL), or both, and then immediately observed in the live cell chamber system under a laser scanning confocal microscope (Carl Zeiss). Live-cell images were acquired every 15 s over a 30-min period and data analysis was performed using ZEN software Blue lite edition (Carl Zeiss).

#### 2.6.2. Measurement of Cellular Antigen Uptake by Flow Cytometry

DC2.4 cells were seeded at a density of 5 × 10^5^ cells/mL in a 24-well plate and cultured overnight. Cells were incubated for 1 h at 4 °C or 37 °C with fresh medium, followed by treatment with fluorescently labeled gE (5 µg/mL), alone or in combination with dLOS (1 or 3 µg/mL), liposomes (50 or 100 µg/mL), or both for 4 h. Endocytosis inhibitors chlorpromazine (50 µM), cytochalasin D (5 µM), or genistein (200 µM) were added to the cells 1 h before sample treatment. Cells were washed three times with PBS and fluorescently labeled gE-associated cells were analyzed by flow cytometry (FACSCanto II; Becton Dickinson, CA, USA). Data acquisition and analysis were performed using FlowJo software (FlowJo, Ashland, OR, USA).

### 2.7. Measurement of Cytokines and Chemokines Secreted from Mouse Immune Cells

J774A.1 cells were seeded at a density of 2 × 10^5^ cells/mL in a 96-well plate, cultured overnight, and treated with liposomes (25, 50, or 100 μg/mL), dLOS (0.5, 1, or 2 μg/mL), or both for 24 h. IL-12 and MCP-1 secreted into the culture medium were measured using cytokine sandwich ELISA kits.

### 2.8. Measurement of Cytokine and Chemokine Secretion at the SOI

Six-week-old female BALB/c mice (SLC) were given an intramuscular injection with dLOS, liposomes, or both in a total volume of 100 μL. Control mice were administered PBS. Muscle tissue was collected from the SOI at various times post-injection and homogenized in organ lysis buffer (R&D systems) containing a protease inhibitor cocktail and phenylmethylsulfonyl fluoride (1 mM) using a homogenizer (IKA, Staufen, Germany). Homogenates were centrifugated at 12,000 rpm for 10 min at 4 °C, and the supernatant was assessed for total protein concentration using a BCA protein assay kit (Pierce, MA, USA). Cytokine and chemokine amounts in muscle lysates were determined by a multiplex assay using Luminex^®^ according to the manufacturer’s instructions (R&D Systems). The panel of cytokines and chemokines included: IFN-γ, IL-10, IL-12p70, IL-17A, IL-1α, IL-1β, IL-2, IL-5, IL-6, IP-10, MCP-1, MIP-1α, MIP-1β, MIP-2, RANTES, TNF-α, and MIG.

### 2.9. Immune Cell Phenotyping of Mouse Muscle Samples

Six-week-old female BALB/c mice (SLC) were given an intramuscular injection with fluorescently labeled gE, alone or in combination with dLOS, liposomes, or both. Control mice were administered PBS. Muscle samples taken from the SOI were chopped into small pieces using scissors and digested with type II collagenase (1 mg/mL) in Hank’s balanced salt solution for 1 h at 37 °C. Cell suspensions were filtered through a 40-µm cell strainer and centrifuged. Cells were resuspended and treated with anti-mouse CD16/CD32 Ab for 10 min to block the FcR, followed by staining with combinations of anti-mouse antibodies: anti-CD11b-FITC, anti-CD11c-FITC, anti-Ly6C-PE, anti-F4/80-PE, anti-Ly6G-PE, anti-MHCII-PE, anti-CD3ɛ-PE, and/or anti-CD11b-PE-Cy7. Immune cell and antigen-positive cell populations of the muscle tissue samples were determined by flow cytometry (FACSCanto II), and data acquisition and analysis were performed using FlowJo software. Flow cytometry gating strategies are presented in Supplementary Figure S1.

### 2.10. In Vivo Imaging Analysis

#### 2.10.1. Fluorescence Imaging Analysis

Five-week-old female BALB/c mice (Orient Bio) in groups of six mice were administered fluorescently labeled gE (5 μg), alone or in combination with dLOS (2 µg), liposomes (100 µg), or both in a total volume of 100 µL via intramuscular injection. At 0, 1, and 24 h post-injection, two mice from each group were anesthetized and monitored for the distribution of the fluorescent dye using an in vivo imaging system (IVIS Lumina II; PerkinElmer Health Sciences, Waltham, MA, USA) with excitation/emission wavelengths of 620 nm/670 nm. The acquisition time was 10 sec per view, and the total radiance on the surface of mice was expressed as photons per second per microwatt per centimeter squared ((p/s)/(μW/cm^2^)).

#### 2.10.2. SPECT/CT Imaging Analysis

For single-photon emission-computed tomography (SPECT)/CT imaging analysis, gE antigen was radiolabeled with ^125^I using pre-coated iodination tubes (Pierce, Rockford, IL, USA) according to the manufacturer’s instructions. Five-week-old female C57BL/6 mice (Orient Bio) were given an intramuscular injection with ^125^I-labeled gE alone (20 µg) or in combination with dLOS (2 µg), liposomes (100 µg), or both in a total volume of 100 µL. SPECT was performed with a four-headed multiplexing multi-pinhole NanoSPECT/CT Plus (Mediso, Budapest, Hungary) using a mouse aperture. An acquisition time of 20 s per view was chosen for SPECT, resulting in acquisition times ranging from 15–20 min per animal. The 7-min CT imaging was performed immediately following the whole-body SPECT imaging at 48-μm resolution. SPECT scans were acquired at 1, 24, 48, and 120 h post-injection. Reconstruction of images was performed using HiSPECT software (Bioscan, Washington DC, USA) without attenuation correction. Reconstructed data from SPECT and CT were co-registered using InVivoScope (Bioscan) for analysis and interpretation.

### 2.11. Measurement of In Vivo Cellular Antigen Uptake and Delivery to Lymph Nodes

Six-week-old female BALB/c mice (SLC) were given an intramuscular injection with fluorescently labeled gE (10 μg), alone or in combination with dLOS (3 μg), liposomes (100 μg), or both. Control mice were administered PBS. Muscle samples and draining lymph nodes were collected at 24 h post-injection. Single cells were prepared from tissue samples and analyzed for antigen-positive cells by flow cytometry.

### 2.12. Antigen-Presenting Cell Assay

To prepare antigen-presenting cells, mouse BMDCs were prepared from 5-week-old female C57BL/6 mice (SLC) as previously described by Han et al. [34] and seeded at a density of 1 × 10^4^ cells/well in a 96-well cell culture plate. Cells were pulsed with VZV gE antigen (2.5 µg/mL) alone or in combination with dLOS (0.5 µg/mL), liposomes (50 µg/mL), or both for 24 h and washed three times with fresh medium. For gE-specific responder cells, CD3^+^ T cells were purified from the spleens of VZV gE-immunized C57BL/6 mice using the magnetic antibody cell sorting (MACS) CD3 MicroBead Kit according to the manufacturer’s instructions (Miltenyi Biotec, Germany). Purified CD3^+^ T cells (1 × 10^5^ cells/well) were added to the pulsed presenter cells at a ratio of 10:1 (T cells:BMDCs) and cultured in a 37 °C CO_2_ incubator. The cell culture medium was collected at 3, 7, and 10 days post-treatment and assayed for IFN-γ and IL-5 by sandwich ELISA.

### 2.13. Statistical Analysis

SPSS 18.0 software (IBM, Armonk, NY, USA) was used for statistical analysis. Differences among the experimental groups were analyzed using one-way ANOVA with Tukey’s multiple comparison test. A two-tailed Student’s *t*-test was used to compare two experimental groups. *P* value of less than 0.05 was considered statistically significant.

## 3. Results

### 3.1. Characterization of Liposomes

**Figure 1:**
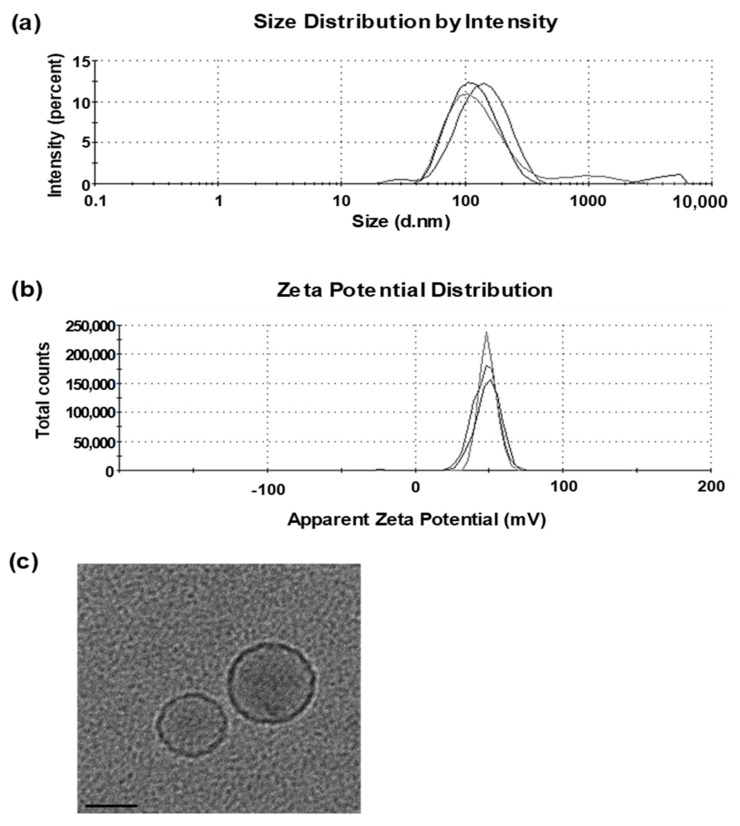
Particle size and morphology of the cationic liposomes. Representative histograms showing the results of three measurements of the particle size (**a**) and zeta potential (**b**) of liposomes. (**c**) Images of liposomes observed under a cryogenic transmission electron microscope (cryo-TEM). Scale bar = 50 nm.

Liposomes prepared using cationic lipid DOTAP and neutral lipid DMPC (1:1, *w*/*w*), had a Z-average diameter of 80–120 nm with a polydispersity index (PDI) of 0.2–0.3 and a zeta potential of + 55–70 mV (Figure 1a,b). The liposomes, observed by cryo-TEM, were mostly small, unilamellar vesicles with a diameter of 50–80 nm (Figure 1c). The addition of dLOS to liposomes did not change the Z-average diameter but tended to reduce the zeta potential of the liposomes by about 10 mV. Since dLOS is negatively charged due to its phosphate moieties, it should interact with positively charged liposomes, thus explaining the decrease in their zeta potential.

**Figure 2:**
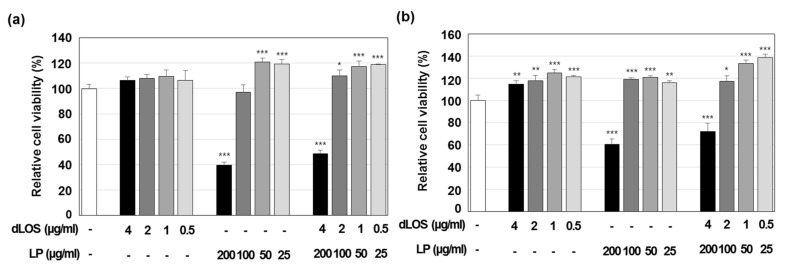
Cytotoxicity of liposomes (LP). MDCK (**a**) and DC2.4 (**b**) cells were incubated with liposomes, dLOS, or both for 24 h, and cell viability was assessed by MTT assays as described in Materials and Methods. Results are expressed as the mean ± SD of values obtained from triplicate assays, and the data shown are representative of at least three experiments with similar results. * *p* < 0.05; ** *p* < 0.01; *** *p* < 0.001 as compared with control cells.

Liposomes were not cytotoxic up to a concentration of 100 µg/mL in either MDCK or DC2.4 cells, but at 200 µg/mL, they killed 40% and 60% of MDCK and DC2.4 cells, respectively (Figure 2). dLOS did not exhibit any cytotoxicity at concentrations up to 4 µg/mL, and its addition to liposomes decreased their cytotoxicity at 200 µg/mL, possibly by lowering the surface charges of the liposomes by their interaction with dLOS. Based on these data, a liposome concentration of 100 µg/mL was used in experiments.

### 3.2. Adsorption of VZV gE Antigen to Liposomes

**Figure 3:**
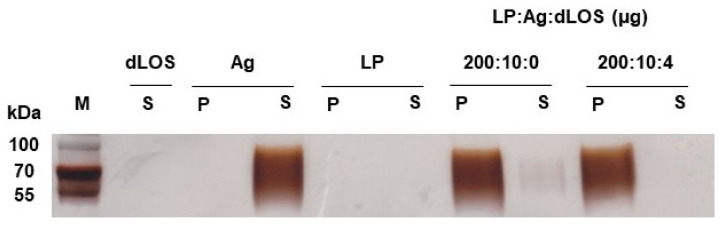
Adsorption of gE antigen to liposomes (LP). VZV gE antigen was mixed with liposomes alone or liposomes plus dLOS and ultracentrifuged. Pellets (P) and supernatants (S) were separated and subjected to SDS–PAGE followed by silver staining. The data shown are representative of two experiments with similar results. M, protein size markers.

Liposomes are thought to promote immune responses by serving as an antigen depot and delivery vehicle. High surface charges on cationic liposomes contribute to adjuvant activity by facilitating antigen adsorption to the liposomes and adsorption of the liposomes to the negatively-charged cell membrane surface [19]. The truncated VZV gE antigen used for the vaccine is negatively charged (pI = 5.5) and is likely to interact with positively charged liposomes [44]. gE was mixed with liposomes and dLOS in the same ratio as in the VZV gE vaccine (liposome:gE:dLOS = 100:5:2, *w*/*w*/*w*) and ultracentrifuged. gE, liposomes, and dLOS controls were included for comparison. After ultracentrifugation, more than 95% of the gE from the liposome mixture, with or without dLOS, was in the pellet, while the gE antigen control was found only in the supernatant (Figure 3). However, the near absence of gE in the supernatant of the mixture of liposomes, gE, and dLOS suggested that the binding of gE to liposomes was more efficient in the presence of dLOS and gE antigen would bind to liposomes in the vaccine formulation.

### 3.3. Enhancement of In Vitro Cellular Uptake of VZV gE Antigen by Liposomes

#### 3.3.1. Liposome-Mediated Cellular Uptake of gE Antigen

**Figure 4:**
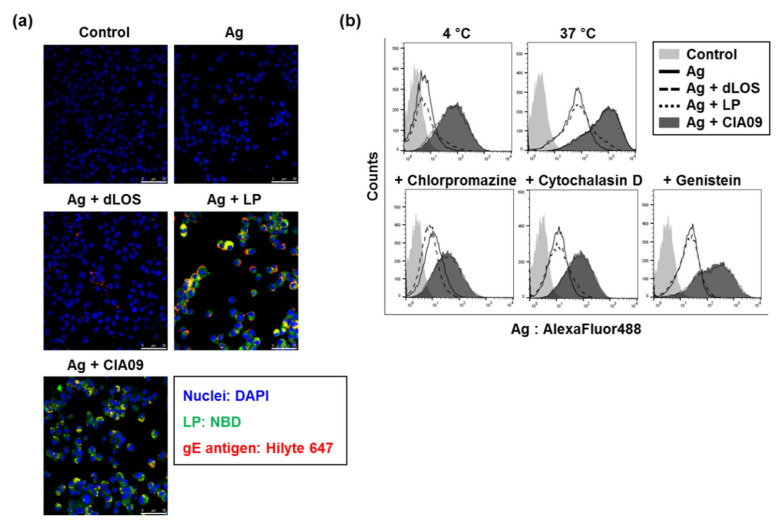
Liposome-mediated antigen uptake by DC2.4 cells. (**a**) Cells were incubated with Hilyte 647-labeled VZV gE antigen alone or gE plus dLOS (3 µg/mL), NBD-labeled liposomes (LP) (100 µg/mL), or both (CIA09) at 37 °C for 4 h, stained with DAPI, and imaged by confocal microscopy. Scale bars = 50 µm. (**b**) Cells were incubated with AF488-labeled gE antigen alone or gE plus dLOS (3 µg/mL), liposomes (100 µg/mL), or CIA09 at 4 °C or 37 °C for 2 h and analyzed by flow cytometry. Endocytosis inhibitors were added to the cells 1 h before sample treatment. The data presented are representative of at least three experiments with similar results.

To determine the effects of liposomes and dLOS on antigen uptake by APCs, we measured gE antigen uptake by DC2.4 cells. Cells treated with Hilyte 647-labeled gE antigen alone showed almost no fluorescence, while the addition of dLOS to gE before treatment slightly increased gE fluorescence in the cells (Figure 4a). The amount of gE greatly increased in cells treated with gE and NBD-labeled liposomes, and the intracellular gE co-located with liposomes, suggesting that gE was tightly associated with liposomes. The amount of gE in cells treated with gE plus CIA09 was similar to cells treated with gE and liposomes, suggesting that dLOS in the CIA09 formulation did not promote antigen uptake by DCs.

To determine whether the gE antigen associated with DC2.4 cells was intracellular, we compared the number of gE-positive cells after incubation of cells with gE plus adjuvants at 4 °C versus 37 °C by flow cytometry (Figure 4b). Incubation of DCs at 4 °C greatly reduced gE levels, especially in cells treated with the antigen plus liposomes or CIA09, indicating that the liposome-associated antigen was taken into cells by an active, temperature-dependent process. Pre-treatment of DCs with endocytosis inhibitors, chlorpromazine, cytochalasin D, or genistein greatly decreased fluorescent cells, indicating that the uptake of liposome-associated antigen was via endocytosis. Consistent with the results obtained by fluorescence microscopy, there was no difference in intracellular gE between liposome- or CIA09-treated cells.

#### 3.3.2. Cooperative Effects of Liposomes and dLOS on the Cellular Uptake of gE antigen

**Figure 5:**
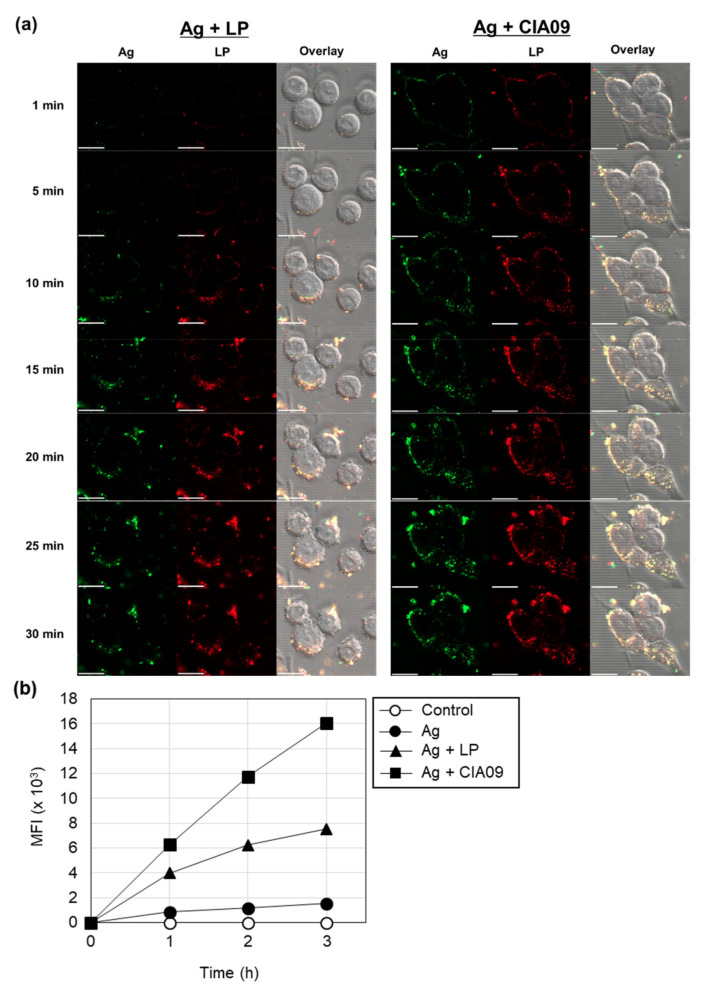
Cooperative effects of liposomes (LP) and dLOS on the cellular uptake of gE antigen. (**a**) DC2.4 cells were treated with AF488-labeled gE antigen (green) in combination with DiR-labeled liposomes (red) or CIA09 and cultured at 37 °C. Cumulative cellular fluorescence was acquired over 30 min using a laser scanning confocal microscope. The data presented are representative of two experiments with similar results. Scale bar = 20 µm. (**b**) DC2.4 cells were treated with AF647-labeled gE antigen, alone or in combination with dLOS (1 µg/mL), liposomes (50 µg/mL), or both, and cultured at 37 °C for 1, 2, or 3 h followed by flow cytometry. The data presented are representative of three independent experiments with similar results.

To determine whether dLOS affected liposome-mediated antigen uptake at short times, we collected confocal images of live cells for up to 30 min for DCs incubated with AF488-labeled gE protein, alone or in combination with dLOS, DiR-labeled liposomes, or with CIA09. In the cells incubated with gE alone or with dLOS, fluorescence was negligible at 30 min, indicating that cellular uptake of gE was minimal (data not shown). In the cells given liposome-associated gE, intracellular antigen was detected after 10 min and gradually increased up to 30 min (Figure 5a). In contrast, for the cells treated with gE and CIA09, antigen uptake began within 1 min, and the intracellular antigen peaked earlier (25 min) and at a higher level than for liposome-treated cells. The Z-stack image analysis of DC2.4 cells showed that both gE and liposomes were co-located in the cytoplasm (data not shown). The antigen uptake assay was repeated using half the amount of liposomes (reducing the influence of liposomes), and cellular gE was measured by flow cytometry after a 1–3 h incubation. CIA09-treated cells had more gE compared to liposome-treated cells, and the cooperative effect of dLOS with liposomes on cellular antigen uptake was evident (Figure 5b). These data demonstrate that liposomes mediate antigen uptake by DCs via endocytosis, while dLOS acts cooperatively with liposomes to promote more rapid internalization of the antigen.

### 3.4. Immune Cell Activation and Recruitment by CIA09

**Figure 6:**
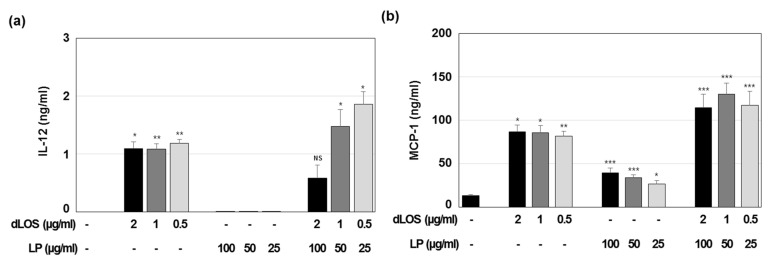
In vitro immune-stimulating activity of liposomes (LP). J774A.1 cells were cultured with liposomes, dLOS, or both for 24 h. IL-12 (**a**) and MCP-1 (**b**) released into the culture medium were measured by sandwich ELISA. Results are expressed as mean ± SD of triplicate cultures. Data presented are representative of at least two experiments with similar results. * *p* < 0.05; ** *p* < 0.01; *** *p* < 0.001 as compared with control cells.

We found previously that dLOS stimulates immune activity in human monocytes as well as in mouse monocyte/macrophage J774A.1 cells and BMDCs [34]. Here, we measured cytokine and chemokine secretion in J774A.1 cells treated with various concentrations of liposomes without dLOS or with dLOS in the same proportion as in the VZV gE vaccine formulation. IL-12 in the culture medium from dLOS-treated cells was about 1 ng/mL, independent of dLOS concentration (Figure 6a). These results were similar to those from our previous studies showing that IL-12 induced by dLOS reached a peak at as low as 0.1 μg/mL of dLOS in these cells (unpublished data). Liposomes alone did not induce cytokine secretion. Neither IL-12 (Figure 6a) nor TNF-α (data not shown) was detected in the liposome-treated cells. Liposomes with dLOS (i.e., CIA09) induced IL-12 secretion in inverse proportion to the adjuvant concentration, suggesting that while liposomes with low concentrations of dLOS enhanced IL-12 release from the cells, at higher concentrations liposomes inhibited the dLOS activity. Although liposomes barely induced cytokines, they stimulated the release of chemokine MCP-1, and that release was enhanced by dLOS (Figure 6b). Liposomes did not induce cytokine secretion or costimulatory surface molecule expression in mouse BMDCs (data not shown).

**Figure 7:**
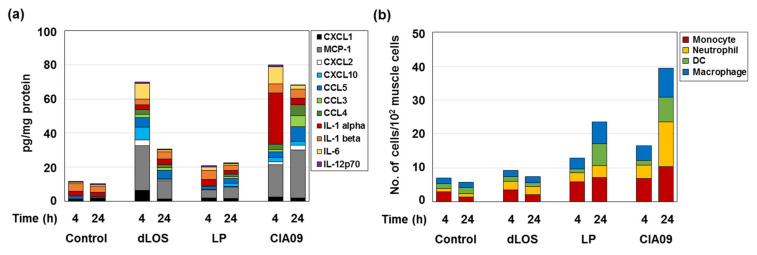
Cytokine and chemokine induction and immune cell recruitment at SOI. Groups of mice (*n* = 3) were given an intramuscular injection with dLOS (3 μg), liposomes (LP) (100 μg), or both (CIA09), and muscle samples were collected at 4 h and 24 h post-injection. (**a**) Muscle tissue homogenates were prepared and analyzed for cytokines and chemokines by multiplex assays using Luminex^®^. (**b**) Single cells were prepared, stained with antibodies specific for immune cell markers, and analyzed by flow cytometry. Data are expressed as the mean of three muscle samples for each group.

Since CIA09 induced cytokine and chemokine release in vitro, we determined its ability to recruit and stimulate immune cells in vivo. Cytokine and chemokine secretion was assayed in mouse muscle tissues 4 h and 24 h after intramuscular injection with dLOS, liposomes, or CIA09 (Figure 7a). dLOS induced strong cytokine and chemokine secretion at 4 h post-injection, which decreased by 50% at 24 h. Liposomes weakly induced cytokine and chemokine secretion and MCP-1was the most abundant species, consistent with the in vitro data (Figure 6). CIA09 was more effective in stimulating the secretion of cytokine and chemokines than dLOS, and high levels of secretion were maintained at 24 h in the CIA09-treated mice but not in the dLOS-treated mice. While there were differences in the cytokine and chemokine profiles of the two groups, both groups had high levels of MCP-1 and IL-6 at 4 h. The predominant species in CIA09-treated mice at 4 h was IL-1α, which decreased to basal level at 24 h.

The high levels of chemokines secreted at the SOI in mice treated with dLOS or CIA09 would be expected to recruit immune cells to the site; however, mice treated with dLOS showed low levels of immune cell recruitment (Figure 7b). In contrast, liposomes increased the frequency of immune cells at 4 h, which doubled at 24 h, while CIA09-treated mice had the largest immune cell population among the groups at 4 h and 24 h. These data suggest that dLOS and liposomes cooperatively activate and recruit immune cells to the SOI.

### 3.5. Increase in Antigen Uptake at the Site of Injection by CIA09

**Figure 8:**
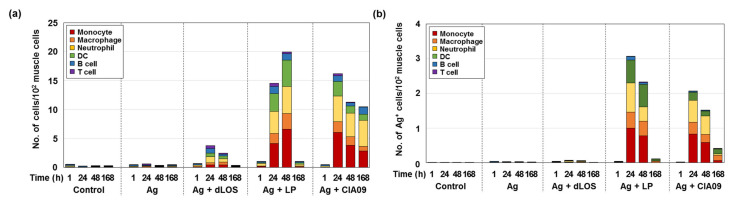
Antigen uptake at the SOI. Groups of mice (*n* = 3) were given an intramuscular injection with AF647-labeled gE (5 μg), alone or in combination with dLOS (2 μg), liposomes (LP) (100 μg), or CIA09, and muscle samples were collected at 1, 24, 48, and 168 h post-injection. Single cells were prepared, stained with antibodies specific for immune cell markers, and analyzed by flow cytometry. (**a**) Immune cell populations at SOI; (**b**) the frequency of antigen-positive immune cells. The results are expressed as the mean of values obtained from three muscle samples for each group. The data shown are representative of three independent experiments with similar results.

To determine whether CIA09 enhances antigen uptake, mice were injected intramuscularly with AF647-labeled gE antigen, alone or in combination with dLOS, liposomes, or CIA09. Muscle samples were collected at 1, 24, 48, and 168 h post-injection and analyzed by flow cytometry for antigen-positive immune cells and total immune cells (Figure 8). Injection of gE antigen did not change the immune cell frequency in muscle cells, while gE antigen with dLOS resulted in only a minimal, temporary increase in immune cells. However, administration of gE antigen with liposomes or CIA09 dramatically increased the immune cell population at the SOI at 24 h post-injection, consistent with the results shown in Figure 7b. While immune cell recruitment reached a peak at 48 h in mice injected with gE plus liposomes and then decreased rapidly to basal level at 168 h, mice administered gE plus CIA09 maintained a high immune cell population up to 168 h post-injection. The antigen-positive immune cell profiles in the muscle samples were similar to the total immune cell population, except that antigen-positive immune cells were greater in mice treated with liposomes compared to CIA09 (Figure 8b). We conclude that liposomes recruit immune cells and mediate their uptake of gE, while dLOS contributes to immune cell retention at the SOI.

### 3.6. Increased In Vivo Stability of Liposome-Associated gE Antigen

**Figure 9:**
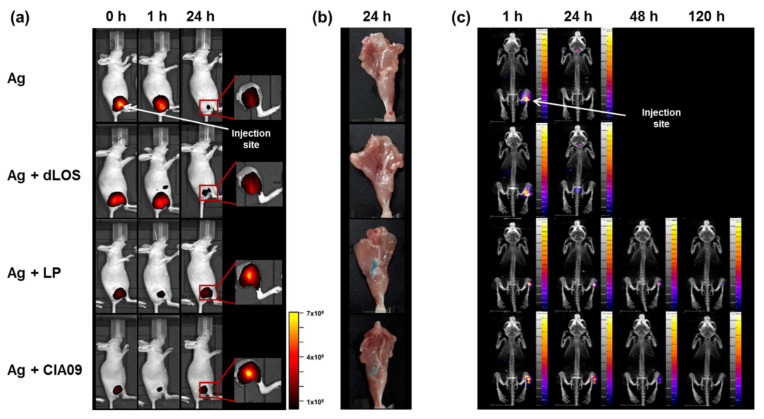
In vivo imaging of CIA09-adjuvanted gE antigen in mice. (**a**) Groups of mice (*n* = 6) were injected with AF647-labeled gE (5 μg), alone or in combination with dLOS (2 μg), liposomes (LP) (100 μg), or CIA09. Two mice from each group were monitored at 0, 1, and 24 h post-injection for the distribution of the fluorescence dye using in vivo imaging. Fluorescence detected at a lower scale are shown in photo inserts. The data shown are representative of two independent experiments with similar results. (**b**) Mouse legs taken at 24 h were opened to monitor fluorescence. An image of one mouse from each group is shown. (**c**) Mice were injected with ^125^I-labeled gE (20 μg), alone or in combination with dLOS (1 μg), liposomes (100 μg), or CIA09, and their SPECT/CT images were obtained at 1, 24, 48, and 120 h post-injection. Arrows mark injection sites.

Since vehicle-type adjuvants such as alum and liposomes form an antigen depot at the SOI, we determined whether liposomes serve as an antigen reservoir at the SOI, increasing antigen stability. The distribution of AF647-conjugated gE after intramuscular injection, alone or in combination with dLOS, liposomes, or CIA09, was monitored at 0, 1, or 24 h post-injection using an in vivo imaging system. A very high level of fluorescent gE was detected immediately after injection at the SOI and at 1 h post-injection (Figure 9a), but decreased by more than 90% after 24 h, indicating that the antigen was quickly degraded. The addition of dLOS to gE yielded results similar to gE alone. However, when gE antigen was mixed with liposomes or CIA09, the fluorescence signal from the gE antigen was reduced about five-fold due to the fluorescence quenching effect of liposomes. Despite this quenching, fluorescence for the group given gE antigen mixed with liposomes or CIA09 at 24 h post-injection was 3–5 times greater than for the group given gE alone (see the inserts of Figure 9a) and was almost the same as the initial level, indicating that the gE antigen at the SOI was retained over time. Postmortem examination of the leg muscles of mice given liposomes or CIA09 revealed a strong blue color of the fluorescently labeled gE antigen, while no trace of color was left in the muscles of the other two groups (Figure 9b).

The in vivo imaging analysis was repeated using ^125^I-labeled gE antigen. The images of the mice injected with ^125^I-labeled gE alone revealed high gE at the SOI one hour after injection but none at 24 h (Figure 9c). ^125^I released from the labeled gE was detected in the thyroid and bladder of these animals. Mice administered ^125^I-gE mixed with liposomes or CIA09 retained large amounts of gE at the SOI at 24 h after injection, with a detectable signal at 48 h that disappeared by 120 h. Interestingly, however, the antigen spread from the SOI in the dLOS-treated mice, probably due to the increased immune cell mobility activated by dLOS. Overall, these findings indicate that liposomes, with or without dLOS, serve as an antigen reservoir and increase the stability of antigen at the SOI.

### 3.7. Efficient Delivery to Lymph Nodes of CIA09-Adjuvanted gE Antigen

**Figure 10:**
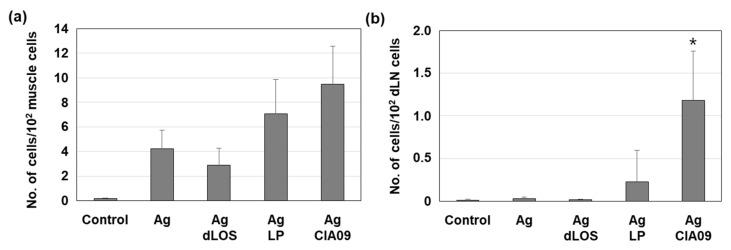
Increased antigen delivery to lymph nodes in the mice administered CIA09-adjuvanted gE antigen. Groups of mice (*n* = 3) were given an intramuscular injection with AF647-labeled gE antigen (10 μg), alone or in combination with dLOS (3 μg), liposomes (LP) (100 μg), or both. Cells were collected from the muscles at SOI (**a**) and draining lymph nodes (**b**) at 24 h post-injection and analyzed for antigen-positive cells by flow cytometry. Results are expressed as the mean ± SD of values obtained from 3 mice for each group. * *p* < 0.05 as compared with the group given antigen alone or antigen plus dLOS.

To determine a possible role of adjuvants in the delivery of antigens to lymph nodes, mice were injected with AF647-labeled gE, alone or with dLOS, liposomes, or CIA09, and the antigen-positive cells at the SOI and in the draining lymph nodes were measured by flow cytometry (Supplementary Figure S2). While the addition of dLOS did not affect the number of gE-positive cells at the SOI compared to antigen alone (Figure 10a), the gE-positive cells increased 1.7 times in mice given gE plus liposomes, although the difference between the two groups was not statistically significant (*p* = 0.48). The addition of CIA09 to gE increased the number of antigen-positive cells at the SOI 1.3-fold compared to the liposome-treated group (*p* = 0.60). There were few antigen-positive cells in draining lymph nodes in mice given gE alone, gE with dLOS, or with liposomes. However, mice injected with CIA09-adjuvanted antigen were significantly higher in antigen-positive cells in lymph nodes compared to those given antigen alone or antigen plus dLOS (*p* < 0.05). Furthermore, they showed a 5.2-fold increase in antigen-positive cells when compared to those given antigen with liposomes, although the difference between the two groups was not statistically significant (*p* = 0.73). These data suggested that dLOS is the primary adjuvant component involved in efficient antigen delivery to lymph nodes, even though both liposomes and dLOS contribute to increased antigen uptake by APCs at SOI. Phenotyping analysis of antigen-bearing immune cells at SOI and lymph nodes revealed that DCs are the primary cells that deliver the antigens to lymph nodes, although macrophages and monocytes are the cells that occupy the most antigens at SOI (Supplementary Figure S3).

### 3.8. Efficient Antigen Presentation to T Cells by DCs Pulsed with CIA09-Associated gE

**Figure 11:**
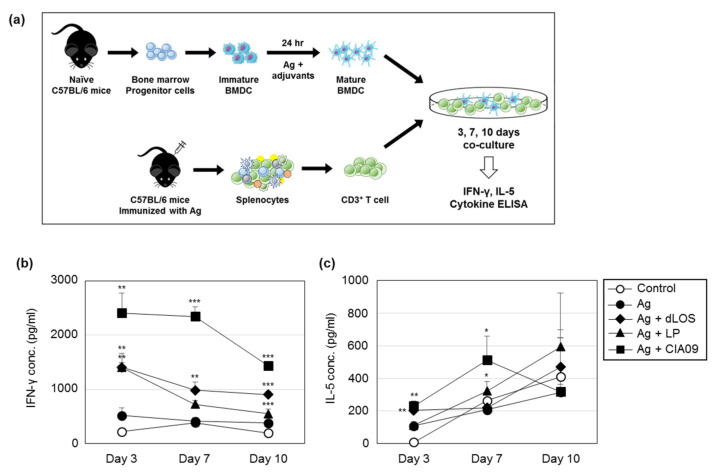
Efficient antigen presentation to T cells by DCs pulsed with CIA09-adjuvanted gE antigen. (**a**) T cells isolated from gE-immunized mice were co-cultured for 24 h with BMDCs that had been pulsed with gE, alone or in combination with dLOS, liposomes (LP), or CIA09. The culture medium was harvested at 3, 7, and 10 days post-treatment and assayed for IFN-γ (**b**) and IL-5 (**c**) by sandwich ELISA. Results are expressed as the mean ± SD of values obtained from triplicate cultures. The data presented are representative of two independent experiments with similar results. * *p* < 0.05; ** *p* < 0.01; *** *p* < 0.001 as compared with antigen-treated cells.

To determine whether CIA09 enhances antigen presentation to T cells, presenter BMDCs isolated from naïve C57BL/6 mice were pulsed with gE alone or in combination with dLOS, liposomes, or CIA09, and co-cultured with syngeneic CD3^+^ T responder cells purified from VZV gE-immunized mice. The culture medium was harvested after 3, 7, and 10 days and assayed for IFN-γ and IL-5 (Figure 11). The amount of IFN-γ secreted by T cells that had been co-cultured with gE-pulsed DCs was very low and similar to the control DCs. When presenter cells were pulsed with gE plus either dLOS or liposomes, there was significantly more IFN-γ secreted by T cells at day 3 (*p* < 0.01), which decreased thereafter. The addition of CIA09 doubled the amount of IFN-γ secreted by T cells at day 3 compared to either dLOS or liposomes (*p* < 0.01), and it remained high until day 10 (*p* < 0.001), indicating efficient antigen presentation by CIA09-pulsed DCs. In contrast to IFN-γ, there was no significant difference in IL-5 between DCs pulsed with gE alone or in combination with liposomes or dLOS. However, DCs pulsed with gE plus CIA09 elicited IL-5 secretion from T cells at day 7, which was significantly greater than the other groups (*p* < 0.05). These data indicate that CIA09-adjuvanted gE is taken up by DCs, which are activated and efficiently present the antigen to T cells.

## 4. Discussion

We have previously developed a VZV gE subunit vaccine for the prevention of herpes zoster that elicited both humoral and cellular immune responses in a mouse model [44]. The vaccine contains an adjuvant formulation CIA09, composed of DOTAP-based cationic liposomes and the TLR4 agonist dLOS, which enhances antibody and cell-mediated immune responses to tuberculosis protein antigens and inactivated JEV vaccine [42,43]. In this study, we investigated the mechanism of action of CIA09 as an adjuvant for the model VZV gE antigen. The two components of CIA09, cationic liposomes and dLOS, cooperate to induce adaptive immunity to vaccine antigens, including antigen uptake by DCs, antigen delivery to lymph nodes, recruitment and activation of innate immune cell to the SOI, and antigen-presentation to T cells.

The protective immunity induced by vaccination depends on the interaction of APCs with T cells, which links innate and acquired immunity, and activation of DCs is essential for antigen processing and presentation to T cells [46,47]. Tissue-resident DCs weakly express surface costimulatory molecules and MHC class II molecules but readily capture antigens from the environment [46,47]. Once activated, DCs lose the ability to take up antigens but express high levels of surface molecules and migrate to draining lymph nodes where they present antigens to T cells [46,47]. Cationic liposomes, such as DDA-based liposomes, can adsorb antigens, enhancing antigen uptake and presentation by APCs [19,23]. In this study, cationic liposomes mediated gE antigen uptake by DCs both in vitro and in vivo, and DCs pulsed with gE plus liposomes efficiently presented gE to antigen-specific T cells leading to IFN-γ secretion. However, the cationic liposomes did not activate surface molecule or cytokine expression in DCs (data not shown). dLOS, a TLR4 agonist, directly activates DCs leading to high levels of CD40, CD80, and CD86; expression of MHC class II costimulatory molecules; and secretion of TNF-α, IL-6, and IL-12 [37]. While dLOS- and MPL-activated DCs induce IFN-γ, IL-5, and IL-17 secretion from T cells in an allogeneic T cell response assay, dLOS is more effective than MPL [37]. In our current study, dLOS promoted antigen uptake by DCs, especially in the presence of liposomes (Figure 5). Liposomes may increase the targeting and uptake of antigens by DCs, while dLOS contributes to the activation of DCs, since CIA09-treated DC cells were activated faster than liposome-treated cells (Figure 5). Generally, CIA09 was more effective than each component alone, suggesting that dLOS and liposomes work cooperatively to present antigen to T cells. The migration of antigen-containing DCs to draining lymph nodes occurred more efficiently in the mice that received antigen plus CIA09 than those administered antigen plus liposomes, supporting the role of dLOS in the activation of DCs. However, the number of mice used in the experiment was too small to see a statistically significant difference between the groups, and the experiment should be repeated using more mice per group in order to draw a definite conclusion.

CIA06, like CIA09, is a dLOS-based adjuvant formulation with aluminum hydroxide instead of liposomes as the antigen carrier [38,39]. CIA06 enhances the serum antibody and CMI responses to vaccines against anthrax, influenza, *Pseudomonas*, and HPV [38,39,40,41], inducing protective antibodies as demonstrated by neutralization assays and challenges in model animals [38,39,40,41]. An HPV vaccine with CIA06 demonstrated the safety and efficacy of CIA06 in humans in a phase I clinical study (unpublished data). Alum is the most commonly used adjuvant in human vaccines with excellent safety and efficacy [48]. It is a strong activator of Th2-type humoral response but tends to inhibit cellular immunity [48]. We found that the alum in CIA06 in HPV VLP and anthrax vaccines suppressed the Th1-type immune response to the antigens induced by dLOS in proportion to the alum dose, consistent with previous reports [38,39]. Unlike alum, liposomes are known to stimulate Th-1 type immunity when used as vaccine adjuvants. Both DOTAP-based and DDA-based cationic liposomes enhance the CMI response to vaccine antigens [42,43,44]. While liposomes alone stimulated the Th1-type response in the antigen-presentation assay, dLOS addition enhanced the effect (Figure 11). CIA06 and CIA09 elicit similar antibody responses to JEV, but CIA09 is superior in its ability to promote CMI responses [42]. The results of this study indicate that the optimal activity of an adjuvant requires an appropriate combination of components acting via different mechanisms; however, CIA09 would be particularly appropriate for vaccines that require a strong CMI response for efficacy.

Understanding the mechanisms of action of adjuvants is crucial for vaccine design, with the potential to allow an assessment of vaccine safety and efficacy at the development stage and at the regulatory level [49,50]. Hauguel and Hackett suggested that vaccine adjuvants can be designed so that the efficacy and toxicity of adjuvants are separated, which would allow adjuvants with optimal efficacy without unintended side effects [51]. Many immunostimulatory adjuvants, especially TLR agonists, act via innate immune receptors and trigger cellular signaling pathways, leading to the production of cytokines such as TNF-α, IL-1β, and IL-6 that may cause inflammation. dLOS, an immunostimulant in CIA09, directly activates innate immune cells, including DCs and monocytes, thereby inducing cytokines that are essential for its adjuvant efficacy [37]. Administration of dLOS via intramuscular injection resulted in high serum levels of cytokines, with particularly high amounts of pro-inflammatory IL-6 and several chemokines (manuscript in preparation). The addition of cationic liposomes attenuated the activity of dLOS, and the serum cytokine and chemokine levels declined 10-fold compared to dLOS alone, potentially preventing a non-specific inflammatory response (manuscript in preparation). While a high concentration of cationic liposomes, which serve as the antigen carrier in CIA09, was cytotoxic to MDCK and DC cell lines (Figure 2), the addition of dLOS reduced the cytotoxicity of liposomes, probably as a result of the decreased surface charge of liposomes interacting with dLOS. We found that a lower ratio of cationic lipids to neutral lipids in liposomes resulted in a lower surface charge of the particles, with decreased stimulation of immune activity and cytotoxicity (data not shown). Overall, our results indicate that a formulation containing two adjuvants from different classes can maximize adjuvant activity through their cooperative effects, while also reducing the potential toxicity of each component.

## Data Availability

Not applicable.

## Supplementary Materials

The following are available online at https://www.mdpi.com/1999-4923/13/3/390/s1, Figure S1: Flow cytometry gating strategy for immune cell phenotyping and antigen-positive cells. Figure S2: Flow cytometric analysis of VZV gE-positive cells in the muscle tissues at SOI and draining lymph nodes. Live cells were gated (FSC/SSC) and analyzed for antigen-containing cells by gating AF647-positive cells. Figure S3: Antigen delivery to lymph nodes in the mice administered VZV gE antigen combined with liposomes or CIA09.
