# Supplementary material for: The Effect of a TLR4 Agonist/Cationic Liposome Adjuvant on Varicella-Zoster Virus Glycoprotein E Vaccine Efficacy: Antigen Presentation, Uptake, and Delivery to Lymph Nodes
Source: Pharmaceutics. 2021 Mar 15;13(3):390. doi: 10.3390/pharmaceutics13030390 (PMC8001429; doi:10.3390/pharmaceutics13030390)
Supplement: Supplementary file 1 [file pharmaceutics-13-00390-s001.pdf]

pharmaceutics

MDPI

Supplementary Materials: The Effect of a TLR4 Agonist/Cationic Liposome Adjuvant on Varicella-Zoster Virus Glycoprotein E vaccine Efficacy: Antigen Presentation, Uptake, and Delivery to Lymph Nodes

Seo Ri Wui, Ara Ko, Ji In Ryu, Eojin Sim, Soo Jeong Lim, Shin Ae Park, Kwang Sung Kim, Ha Kim, Hyewon Youn and Na Gyong Lee*

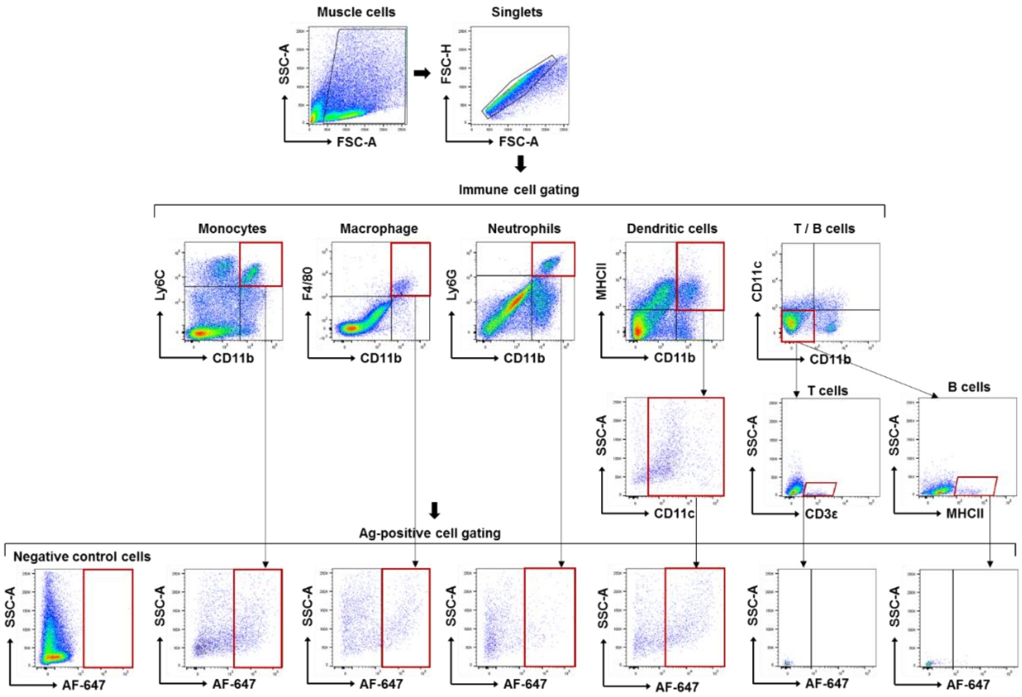

Figure S1. Flow cytometry gating strategy for immune cell phenotyping and antigen-positive cells. A total of 50,000 cells were acquired, and live cells were gated according to forward scatter (FSC) and side scatter (SSC) followed by gating of each immune cell type. Immune cell types were defined as cell surface markers for monocytes (CD11b$^+$ Ly6C$^+$), neutrophils (CD11b$^+$ Ly6G$^+$), dendritic cells (CD11b$^+$ MHCII$^+$ CD11c$^+$), macrophages (CD11b$^+$ F4/80$^+$), B cells (CD11b$^-$ CD11c$^-$ MHCII$^+$), and T cells (CD11b$^-$ CD11c$^-$ CD3$^+$). AF647-positive cells were gated for antigen-bearing cells. Negative control cells were obtained from control mice administered PBS.

Publisher's Note: MDPI stays neutral with regard to jurisdictional claims in published maps and institutional affiliations.

Copyright: © 2021 by the author. Licensee MDPI, Basel, Switzerland. This article is an open access article distributed under the terms and conditions of the Creative Commons Attribution (CC BY) license (http://creativecommons.org/licenses/by/4.0/).

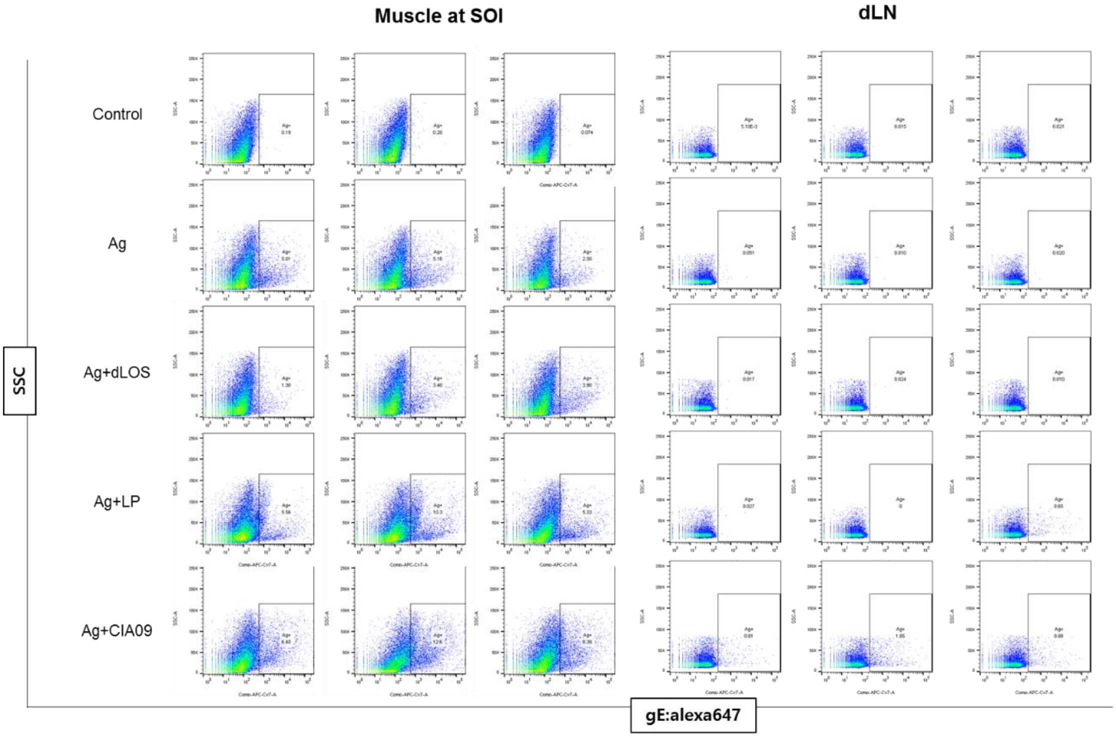

Figure S2. Flow cytometric analysis of VZV gE-positive cells in the muscle tissues at SOI and draining lymph nodes. Live cells were gated (FSC/SSC) and analyzed for antigen-containing cells by gating AF647-positive cells.

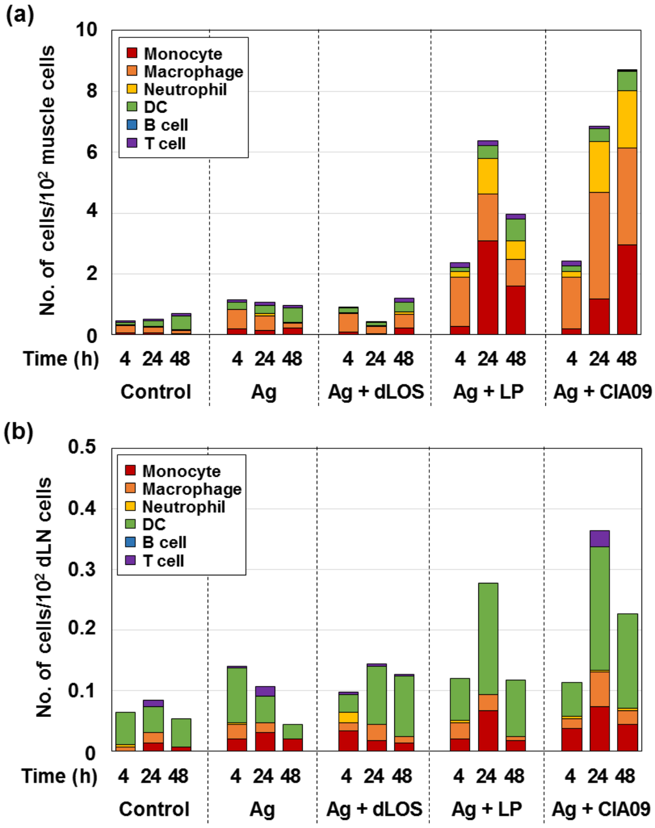

Figure S3. Antigen delivery to lymph nodes in the mice administered VZV gE antigen combined with liposomes or CIA09. Groups of mice ($n = 3$) were given an intramuscular injection with AF647-labeled gE antigen (5 μg), alone or in combination with dLOS (3 μg), liposomes (LP) (100

μg), or both. Cells were collected from the muscles at SOI (a) and draining lymph nodes (b) at 4, 24, and 48 h post-injection and analyzed for immune cells containing antigen by flow cytometry. Results are expressed as the means of values obtained from 3 mice for each group.
